# Dietary Plant-Based Protein Supplements: Sources, Processing, Nutritional Value, and Health Benefits

**DOI:** 10.3390/foods14183259

**Published:** 2025-09-19

**Authors:** Kartik Sharma, Wanli Zhang, Saroat Rawdkuen

**Affiliations:** 1 Unit of Innovative Food Packaging and Biomaterials, School of Agro-Industry, Mae Fah Luang University, Chiang Rai 57100, Thailand; kartik.coa@gmail.com; 2 School of Food Science and Engineering, Hainan University, Haikou 570228, China; zwl@hainanu.edu.cn

**Keywords:** protein supplements, sources, sustainable, novel processing technologies, health benefits

## Abstract

With the global population reaching 10 billion in 25 years, food production must increase 70% while addressing sustainability concerns. This review uniquely integrates advanced processing technologies—including precision fermentation, AI-driven optimization, and 3D printing—with comprehensive analysis of nutritional quality and health outcomes of plant-based protein supplements (PBPSs). Common sources include legumes, cereals, and nuts/seeds, each with amino acid profiles requiring strategic protein complementation. Advanced processing technologies including high-pressure processing, ultrasound-assisted extraction, pulsed electric field, precision fermentation, and AI-driven optimization enhance protein digestibility, solubility, and functional properties while reducing antinutritional factors. PBPSs demonstrate comparable muscle protein synthesis to animal proteins while providing superior cardiovascular, metabolic, and gut health benefits due to bioactive compounds, fibers, and antioxidants. Integrating advanced processing with traditional methods presents opportunities to develop high-quality, sustainable protein supplements meeting global demands while promoting human health and environmental sustainability.

## 1. Introduction

**Figure 1:**
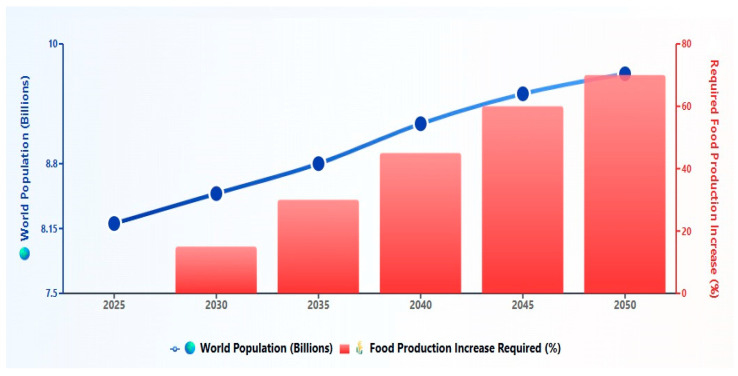
Global population growth projections and corresponding food production requirements from year 2025 to 2050. Source: [2,3].

Proteins are naturally occurring biopolymers of amino acids which play an important role in maintaining human health across all stages of life. In the food industry, proteins are used as a backbone to design different nutritious diets that meet daily nutritional requirements. They are considered as the major source of nitrogen in the diet and aim to provide essential amino acids required for building and maintenance of tissues. In simple words, amino acids or proteins are known as the building blocks of the body. In addition, some amino acids must be obtained from the diet for synthesis of physiological enzymes, which regulate chemical and biological reactions in the body and are essential for sustaining normal body activity [1]. Figure 1 depicts the projected global population growth and corresponding increases in food production needs from 2025 to 2050. These projections illustrate the urgent need for alternative protein sources to meet rising demands sustainably.

It is expected that the global population will reach 10 billion within the next 25 years, which requires an additional 70% increase in food production to meet the corresponding rise in demand [4]. The growing demand of food is not solely due to the rising population but also stems from increasing consumer awareness of sustainability and environmental concerns, thereby causing heightened interest in alternative protein sources. While animal-derived proteins are nutritionally valuable, their production is associated with significant environmental burdens [5]. For example, the production of 1 kg of high-quality animal protein requires feeding of 6 kg plant protein to livestock, which introduces subsequent strain on land and water resources, as well as potential increases in greenhouse gas emissions, associated with livestock agriculture [5]. These adverse environmental effects upon producing animal proteins have led to the search for different alternative protein sources, including plant-based proteins, marine life, insects, and microbial protein. Furthermore, consumer concerns about the high environmental impact from animal proteins coupled with changes in food preferences and dietary patterns, particularly in the Western countries, have led to a gradual shift toward alternative protein sources, including the plant-based protein supplements (PBPSs) [1]. These alternative sources of proteins are also gaining attention due to their low cost, reduced environmental impact, and nutritional benefits [6]. Expanding the usage of plant-based proteins offers a practical way to meet the growing demand for high-quality protein and lessen the environmental damage caused by traditional animal protein production [1].

**Figure 2:**
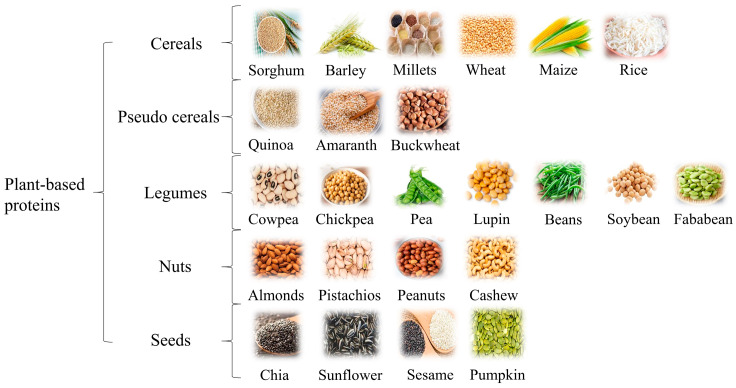
Variety of plant-based protein sources.

As the name suggests, plant-based proteins are those that originate from a diverse array of botanical sources, including cereals, pseudocereals, legumes, seeds, and whole grains, as illustrated in Figure 2 [7,8]. While these sources may be deficient in one or more micronutrients, such as vit B12, vit D, and iron, they are rich in particular amino acids that can be easily absorbed by the body. They are generally considered healthy, as they are rich in polyunsaturated fatty acids, dietary fibers, carbohydrates, and oligosaccharides [8]. As a result, plant-based proteins have been associated with positive health outcomes, such as a lower incidence of type II diabetes mellitus, a lower risk of cardiovascular diseases, a lower level of low-density lipoprotein (LDL) cholesterol, and a lower rate of obesity [8]. As shown in Figure 2, plant-based proteins are derived from a wide range of botanical sources, including cereals, legumes, pseudocereals, seeds, nuts, and whole grains.

Given the increasing interest in sustainable and health-promoting dietary solutions, there is a pressing need to better understand the potential of plant-based protein supplements in modern nutrition. While previous reviews have addressed individual aspects of PBPSs, there remains a significant gap in comprehensively integrating advanced processing technologies (including novel biotechnological approaches like precision fermentation, AI-driven optimization, etc.) with their direct impacts on nutritional quality and health outcomes. The majority of the literature addresses individual protein sources or standalone processing methods, without presenting an integrated framework that elucidates how these technological advancements address the inherent limitations of plant proteins. Therefore, the objective of this review is to fill this gap by comprehensively exploring the sources, production methods, quality attributes, nutritional composition, and health benefits of dietary PBPSs.

## 2. Sources of Plant-Based Protein Supplements (PBPSs)

PBPSs exhibit diverse compositional profiles, functional roles, and processing challenges. The protein content and amino acid composition significantly influence their digestibility, bioavailability, and suitability for incorporation into various food matrices. A comprehensive understanding of these characteristics is essential for optimizing product formulation and addressing the nutritional requirements of diverse consumer populations.

### 2.1. Legumes (Soybeans, Peas, Lentils, Chickpeas)

Legumes are fundamental to plant-based diets, providing an abundant source of protein, carbohydrates, fiber, and essential micronutrients such as iron, zinc, calcium, and magnesium. Their protein content typically ranges from 17% to 40% of dry weight, a level comparable to that found in meat [9]. Beyond proteins, legumes are rich in monounsaturated and polyunsaturated fatty acids, and both soluble and insoluble dietary fiber [10]. The consumption of legumes is associated with a reduced risk of various chronic diseases, including cardiovascular disease (CVD), obesity, high blood pressure, dyslipidemia, and type 2 diabetes (T2D) [11]. Furthermore, legumes can mitigate oxidative stress, reduce inflammation, and promote gut microbiota diversity. However, legumes contain antinutritional factors, such as phytates and tannins that can interfere with nutrient absorption [12]. These limitations can be significantly mitigated through various processing techniques like soaking, germination, fermentation, and heat treatments, which enhance protein bioavailability and amino acid availability [13]. Legumes are typically rich in lysine but are often limited in sulfur-containing amino acids [14]. As summarized in Table 1, legumes like soybeans and chickpeas are high in protein and rich in key micronutrients despite having some limiting amino acids.

**Soybeans:** Soy protein is known for its high nutritional quality, boasting a Protein Digestibility-Corrected Amino Acid Score (PDCAAS) of 1.00, comparable to high-quality animal proteins like milk, whey, and egg protein [5]. It is rich in indispensable amino acids, due to the presence of all nine indispensable amino acids, although it can be slightly limited in sulfur-containing amino acids such as methionine and cysteine.

Nonetheless, soy protein is notably rich in conditionally indispensable amino acids like arginine, glutamine, and glycine, which are vital for functions such as the urea cycle and collagen synthesis. Nutritionally, soybeans offer soluble fibers, oligosaccharides, B vitamins, minerals, soy lecithin, and bioactive phytoestrogens (isoflavones) [15]. They are low in saturated fat and serve as a source of unsaturated and omega-3 fatty acids.

**Figure 3:**
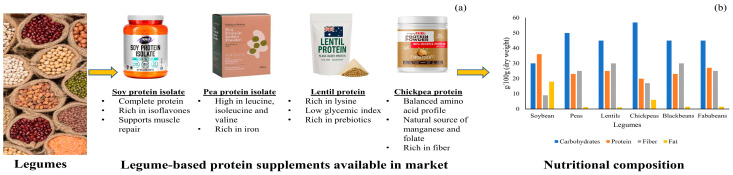
(**a**) Commercial formulations of legume-based protein powders; (**b**) macronutrient comparison of commonly consumed legumes.

Consumption of soy protein is linked to improved cardiovascular health (by lowering LDL cholesterol), reduced metabolic syndrome risk, and potential benefits in managing menopause and hormone-dependent cancers (breast and prostate) [16]. Although the acute muscle protein synthesis response to soy may be slightly lower than whey, soy supplementation has shown comparable improvements in lean body mass and muscle strength in resistance-trained individuals, especially when leucine intake is sufficient. Figure 3 depicts the commercial formulations and nutritional comparison of legume-based proteins, highlighting soy’s superior protein content.

Despite its advantages, soy faces challenges related to off-flavors and antinutrients; however, these can be reduced through appropriate processing [17]. Additionally, soy is frequently used in food fortification and is one of the most industrially produced plant proteins worldwide.

**Peas:** Pea protein is a popular choice for plant-based supplements, often found in blends with rice protein. Pea seeds typically contain 20–25% protein on a dry weight basis [18]. Pea protein isolate (PPI) is its most common supplement form. While generally low in methionine, pea protein has demonstrated significant benefits in promoting muscle thickness, mass, strength, and power gains during resistance training, with results comparable to whey and soy proteins [19]. It has also been shown to reduce postprandial glycemic responses and contribute to satiety.

Pea protein is highly versatile, used in products like eggless cakes and dairy alternatives. When purified from antinutritional factors to its simplest form, such as pea protein isolate, it can exhibit a digestibility rate similar to animal-based proteins [20].

**Lentils and Chickpeas:** These legumes are also important protein sources, with lentils generally being low in methionine. Replacing red meat with legumes like lentils and chickpeas can significantly decrease fasting blood glucose, insulin, and triglyceride levels [8]. The protein content in chickpeas can vary substantially depending on the form, from 20% in flour to 98% in concentrate [7]. However, whole-legume forms tend to have lower protein absorbability due to fiber and other matrix components.

A post-exercise meal consisting of lentils has shown favorable effects on fat oxidation compared to potatoes and eggs, attributed to the low glycemic index of lentils [19]. As with other legumes, lentils and chickpeas contain antinutritional factors such as phytates and tannins, but these can be effectively reduced through processing techniques like soaking, germination, and fermentation. Further discussion of antinutrients appears in the digestibility section.

### 2.2. Cereals (Rice, Quinoa, Oats)

**Figure 4:**
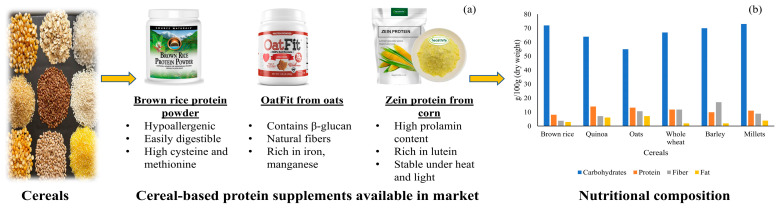
(**a**) Commercial formulations of cereal-based protein powders; (**b**) macronutrient comparison of commonly consumed cereals.

Cereals are another crucial category of PBPSs, though they often exhibit specific amino acid deficiencies. They are commonly low in lysine, tryptophan, and threonine. To overcome these limitations, cereals are frequently combined with legumes in protein blends to achieve a more complete amino acid profile, a strategy known as protein complementation. Figure 4 demonstrates the commercial formulation and macronutrient profiles of major cereal-based protein sources.

**Rice protein**, especially that from brown rice, is valued for its hypoallergenic properties, making it a suitable alternative for individuals with sensitivities to dairy or soy. Its amino acid profile includes glutamic acid, aspartic acid, leucine, arginine, valine, alanine, phenylalanine, and tyrosine. Rice protein has been shown to stimulate muscle protein synthesis at levels comparable to whey protein. Research also suggests that rice endosperm protein can help slow the progression of fatty liver and diabetic nephropathy [20]. Rice protein is widely used in blends and commercial supplements, and due to increasing demand, it is also a subject of adulteration testing to ensure authenticity.

**Quinoa**, though botanically a pseudocereal, stands out among cereals for being a complete protein, containing all nine indispensable amino acids. Its protein content ranges from 12% to 23% [21]. Quinoa is particularly rich in lysine and methionine and is naturally gluten-free, making it a valuable option for individuals with celiac disease. It is utilized in supplements and blends and has shown promising health benefits, such as improving clinical features and metabolism of non-alcoholic fatty liver disease in animal models when incorporated in functional food systems like yogurt.

**Oat protein**, while not extensively studied, is used in supplements and blends and has demonstrated beneficial effects on muscles during recovery. Its amino acid composition along with its β-glucan content demonstrates potential benefits in muscle recovery and makes it a supportive protein source in blended supplements [22].

Although cereals may be limited in some essential amino acids, they offer various health-promoting components such as complex carbohydrates, fiber, and bioactive compounds, making them suitable for complementary use in plant-based protein formulations. Table 1 also illustrates the amino acid limitations of cereals and how their micronutrient content compares with other PBPSs.

**Rice bran protein (RBP)**, obtained from defatted rice bran, represents an underutilized but promising cereal protein source derived from rice processing by-products. RBP contains 10–15% protein with excellent nutritional quality, including well-balanced amino acids and hypoallergenic properties. Esmaeili et al. [23] demonstrated that RBP solubility varies significantly with pH, showing enhanced solubility under alkaline conditions (pH 10–12: 80–82%) compared to acidic conditions (pH 4.0: 8%). This pH-dependency directly affects the functional properties, including emulsifying and foaming capacities. However, recent research has revealed significant processing limitations that constrain RBP applications. Zhao et al. [24] demonstrated that rice bran rancidity significantly affects protein structural characteristics and rheological properties, with storage-induced lipid oxidation causing protein structural changes that compromise functional properties through altered fibril aggregate formation. Mohammadi et al. [25] investigated RBP rheological behavior, revealing distinct limitations compared to conventional proteins, though showing potential in specific food applications such as dairy desserts with careful processing optimization. Furthermore, compared with animal proteins, plant proteins—including cereal-derived fractions such as RBP—are constrained by lower digestibility and limiting indispensable amino acids, antinutritional factors (e.g., phytate, tannins, protease inhibitors) that reduce bioaccessibility, solubility/gelation gaps near neutral pH that yield weaker networks, and sensory off-notes. Recent advances include composite protein systems, where Zhang et al. [26] combined RBP with sodium caseinate to achieve enhanced foaming capacities (177.50%) and improved stability (80.28%), demonstrating strategies to overcome traditional RBP functional limitations while maintaining its hypoallergenic and nutritional benefits. Also, various studies highlight process-enabled solutions such as fermentation, targeted enzymatic modification/cross-linking, and physical treatments (e.g., high pressure, ultrasound) that can raise the DIAAS and improve techno-functionality while managing flavor [27,28].

### 2.3. Nuts and Seeds (Hemp, Pumpkin, Almonds, Chia Seeds)

Nuts and seeds are nutrient-dense sources of plant protein, rich in fiber, healthy polyunsaturated and monounsaturated fats, vitamins, and minerals. While some nuts and seeds, such as those from the almond, may be relatively low in lysine, their overall protein content is significant, making them valuable components of PBPSs.

**Hemp seeds** possess an exceptional nutritional profile and offer various health benefits. Hemp protein supplements, which can contain up to 49% protein, have shown the highest individual phenolic content and total phenolic content (TPC) among tested plant-based protein supplements, indicating strong antioxidant activity [29]. Studies have also confirmed that hemp protein supplementation effectively improves muscle mass, strength, and power gain during training. Hemp protein, widely used in plant-based supplements and blends, is also recognized as a sustainable protein source.

**Pumpkin seeds** are another high-protein option, with some concentrates having up to 65% protein, whereas true isolates generally exceed 80% protein. These proteins exhibit notable antioxidant activities and are used in PBPSs and blends [29]. Pumpkin seeds are a valuable source of protein, which can help in eradicating protein malnutrition, and lipids (rich in PUFAs), containing essential as well as non-essential fatty acids, which prevent various ailments like cancer and other cardiovascular diseases. Since seeds of pumpkin are abundant in macro- (magnesium, phosphorous, potassium, sodium, and calcium) and microminerals (iron, copper, manganese, zinc, and selenium), they can be used as an incredible dietary supplement which in turn helps in curing various deficiency disorders.

**Almonds** are utilized in the production of non-dairy milk alternatives, such as almond milk and cheese-like products. While lower in lysine, almonds still contribute protein, fiber, and beneficial fats. Almonds are a good source of plant-based protein, providing about 10–27 g protein per 100 g [30]. This protein content makes them a beneficial addition to a balanced diet, particularly for those seeking plant-based protein sources, according to eatingwell.com and verywellfit.com. Almonds also offer other nutrients like healthy fats, fiber, and vitamin E [31].

**Figure 5:**
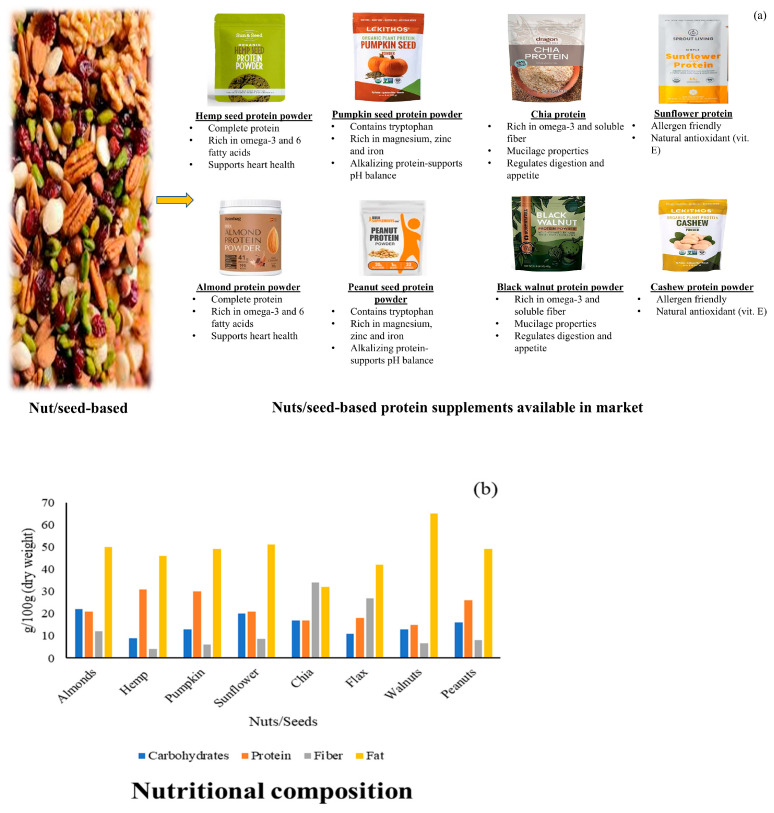
(**a**) Commercial formulations of nut- and seed-based protein powders; (**b**) macronutrient comparison of commonly consumed nuts and seeds.

**Chia seeds**, flaxseeds, and sunflower seeds are recognized as protein sources, contributing to the diversity of plant-based protein options. Sunflower meal protein isolate enhances baking performance in functional foods, while chia and flax provide both amino acids and omega-3 fatty acids [29]. Figure 5 presents the various commercial formulations and nutritional attributes of nut- and seed-based protein supplements. Table 1 summarizes the comparative nutritional composition of commonly used PBPSs, including protein content, digestibility scores, limiting amino acids, key micronutrients, and functional attributes.

**Sesame (*Sesamum indicum* L.)** protein represents a highly promising plant protein source with exceptional nutritional quality and unique functional properties. Ghorbani et al. [32] demonstrated that sesame protein isolate (SPI) exhibits high protein extraction yield (77.2%) and excellent protein content (90.60%), with a well-balanced essential amino acid profile that distinguishes it from other plant proteins. The study revealed optimal pH-dependent functional properties, with peak solubility and functional characteristics at acidic and alkaline pH levels, while showing minimal activity at the isoelectric point (pH 5–5.5). Recent advances in non-thermal processing have significantly improved sesame protein functionality, with Gul et al. [33] showing that high-intensity ultrasound treatment enhanced solubility, reduced particle size, and improved emulsifying and foaming properties compared to untreated controls. Additionally, modified sesame protein isolate has shown effectiveness in preventing phase separation in food applications, demonstrating its industrial potential. Rafe et al. [34] investigated dynamic rheological properties of sesame protein dispersions, revealing unique viscoelastic behavior with good gel-forming capacity, though requiring careful pH and concentration optimization for optimal food applications.

In summary, PBPS sources exhibit complementary nutritional profiles that can be strategically combined to overcome individual limitations. While legumes provide high protein content but lack sulfur-containing amino acids, cereals offer methionine but are limited in lysine. Nuts and seeds contribute healthy fats and unique bioactive compounds. This diversity enables protein complementation strategies to achieve complete amino acid profiles comparable to animal proteins.

**Table 1:** Comparative nutritional composition of commonly used plant-based protein sources, including protein content, digestibility scores, limiting amino acids, key micronutrients, and functional attributes.

Source	Protein (g/100g)	PDCAAS Score	DIAAS Score	Limiting Amino Acid	Key Micronutrients	Notable Features	Reference
Cereals							
Wheat	Flour: 8–15%; seitan: 20.93% (db)	0.45–0.54	0.39	Lysine, isoleucine, leucine, aromatic amino acid, threonine, and valine	Magnesium, molybdenum, zinc, calcium	Increases muscle protein synthesis. Often used in meat imitation products.	[5,35]
Oats	45–55.4% (oat protein concentrate)	0.77 (oat protein concentrate)	0.44	Lysine and threonine	Iron, zinc, manganese	Used to attenuate glycemic response of sugar-sweetened beverages.	[36]
Corn (whole-grain maize)	13.4%	0.41–0.50	0.38	Lysine (primarily), also isoleucine, sulfur-containing amino acids, threonine, and tryptophan	Magnesium, zinc, vitamins B1 and B6	Tends to meet methionine content requirements.	[5,37]
Barley	11%	0.50–0.76	0.50	Lysine	Boron, calcium, copper, iron, potassium, magnesium, manganese, phosphorus, zinc	Barley has a high mean fecal true protein digestibility.	[5,19,38]
Buckwheat	5.67%	NA	0.33–0.56	Threonine and leucine	Vitamins B1, B2, B5, B6, vitamin C, vitamin E	Rich in lysine. It is gluten-free, making it an alternative for patients with celiac disease.	[38,39]
Quinoa	12–23%	0.75	0.68	-	Vitamin A, vitamins B1, B2, B5, B6, B9, vitamin C, vitamin E	Improved clinical features and metabolism of high-fat diet-induced non-alcoholic fatty liver disease in mice.	[38,40,41]
Rice	70–78 (rice protein concentrates or isolates)	0.50–0.60	0.29–0.42	Brown rice low in lysine	B vitamins, selenium	Easy to digest. Increases muscle mass and strength. Rich in methionine. Rice protein is applied in nanomaterial synthesis.	[42]
Legumes							
Faba bean	20–35% (db)	0.60–0.67	0.77	Primarily methionine, histidine, and tryptophan	Iron, zinc, folate	Promising protein source. Faba bean protein concentrates and isolate showed positive effects on postprandial glycemia and appetite.	[43,44]
Soy protein isolate	88–90	0.92	0.68–0.97	Sulfur	Iron, calcium, magnesium	Complete protein, rich in isoflavones.	[42]
Pea protein concentrate	20% in flour to 98% in concentrate	0.72	0.82	Sulfur and tryptophan	Phosphorus, iron, zinc	Hypoallergenic, rich in arginine.	[45]
Lentils	27% in flour to 91% in lentil protein concentrate	0.68–0.80	0.75 (whole food)	Primarily methionine, and lysine	Iron, zinc, calcium, magnesium	Highly nutritious legume that can enhance protein intake in food products.	[5]
Common beans (mung bean, broad bean, kidney bean)	20–25%	0.50–0.65	0.54–0.61	Methionine, cysteine, and tryptophan	Iron, folate, magnesium, potassium	Rich source of carbohydrates, protein, energy, vitamins, minerals, and fibers.	[46,47]
Nuts/seeds							
Hemp	50–60	0.51	0.54	Lysine	Omega-3, zinc, magnesium	Balanced omega-6:3 ratio.	[45]
Chia	35–40	0.65	-	Lysine	Calcium, omega-3, phosphorus	High in fiber and antioxidants.	[41,48,49]
Almonds	10–35	0.35	0.40	Lysine	Iron, zinc, calcium	Good source of dietary fibers.	[5,50]
Sesame	50–60	0.42–0.48	0.45	Lysine and methionine	Calcium, magnesium	Good source of healthy fats, antioxidant properties.	[32,33]

Note: PDCAAS: Protein Digestibility-Corrected Amino Acid Score; DIAAS: Digestible Indispensable Amino Acid Score; db: dry basis.

## 3. Product Forms of Plant-Based Protein Supplements: Ingredients and Formulation

**Figure 6:**
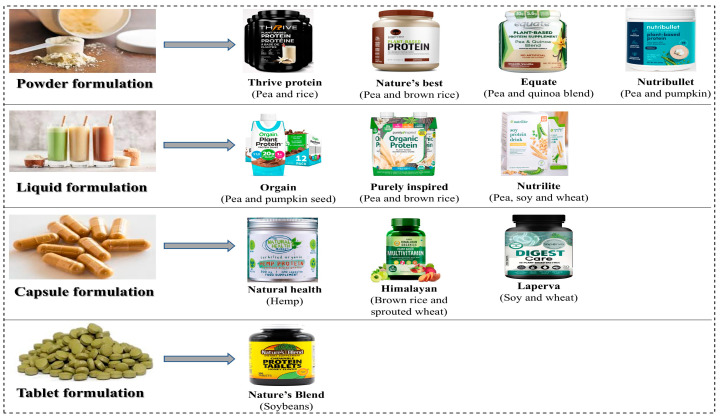
Commercial product forms and formulations of plant-based protein supplements: powders, liquids, capsules, and tablets.

The diverse commercial product forms available for plant-based protein supplements is shown in Figure 6. The production of PBPSs offers promising solutions for diverse consumer needs, nutritional requirements, and current lifestyle preferences. The heightened consumer awareness of and growing demand for clean label products have led to increased production, distribution, and consumption of PBPSs. Understanding the various commercial product forms is essential for optimizing bioavailability, therapeutic efficacy, and consumer compliance across different application contexts.

### 3.1. Powder-Based Formulations

Powder forms are considered as the most commercially available forms of PBPSs due to their processing flexibility and versatile application potential. Various protein concentrates (70–89% protein) and isolates with protein content ≥90% (by weight) are produced via advanced fractionation techniques, effectively removing lipids, carbohydrates, and other antinutritional factors. The final refined product obtained via this method exhibits high solubility, improved digestibility, and reduced antinutritional factors, when compared with whole protein [8]. Protein concentrates, in comparison to protein isolates, possess higher fiber components and micronutrients and thus can be employed for broader commercial applications. On the other hand, protein isolates exhibit superior amino acid bioavailability due to lower availability of antinutritional factors, making them suitable for sports and other therapeutic applications [5].

In addition, multisource protein blending, where a deficient source is combined with complementary protein, is another approach to address the essential amino acid limitation of individual plant sources. For instance, combining legumes (rich in lysine) and cereals (rich in methionine) results in a more complete and balanced profile of essential amino acids. Research suggests that upon accurate strategic blending of different proteins, amino acid scores comparable to those of animal proteins can be attained [1]. Protein powders, concentrates, and isolates differ in solubility and nutrient retention, as shown in Table 2.

### 3.2. Liquid-Based Formulations

Liquid formulations often prove advantageous in terms of enhanced amino acid absorption and fast gastric emptying. Liquid formulations face challenges in maintaining protein stability in aqueous systems, and therefore requires careful pH optimization, ionic strength, and careful selection and incorporation of stabilizing agents. Heat treatment is another crucial factor which needs to be considered during processing, as it induces protein denaturation or aggregation, resulting in lower bioavailability.

Technologies such as precision fermentation, extrusion, scaffolding, etc., are often employed by food industries with the aim of exploring and developing products from plant-based sources. Incorporation of plant proteins into functional beverages offers excellent opportunities to develop beverages enriched with bioactive compounds and essential micronutrients. This approach results in synergistic nutritional profiles that go beyond simple protein supplementation; however, it requires careful consideration of ingredient interactions, bioavailability optimization, and sensory compatibility [29]. Beverage formulations offer immediate bioavailability but face challenges in stability, as summarized in Table 2.

### 3.3. Tablet- and Capsule-Based Formulations

Solid dosage forms such as tablets and capsules minimize the issues related to taste, thereby enhancing the overall consumer acceptability. However, these formulations have lesser protein per unit when compared to other formulations like powders or beverages. Therefore, higher doses of tablets or capsules are required to achieve adequate protein intake. Furthermore, careful designing and monitoring of manufacturing processes (compression and encapsulation) should take place to ensure protein stability and optimal dissolution, and support bioavailability [13].

Other formulations include protein bars or structured products, in which the plant protein is incorporated into solid matrices. Such processes also require careful selection of binding agents and moisture management in order to prevent texture degradation upon storage [51]. In addition, functional food integration, involving protein-fortified food products and target delivery systems, provides an excellent opportunity for increasing the nutritional value of staple foods. Table 2 presents an overview of commercial PBPS product forms and their nutritional efficiency, formulation challenges, bioavailability characteristics, and application contexts.

### 3.4. Applications of Plant-Based Protein in Food Systems

Plant proteins are deployed as structure-directing ingredients across emulsions, foams, gels, beverages, and meat/dairy-analog matrices, supplying interfacial stabilization, water/oil binding, and rheology control in place of animal proteins [52]: in fat-mimetic systems, plant-based emulsion gels/HIPEs deliver spreadable textures and thermal stability via protein–polysaccharide structuring and particle-stabilized interfaces [9,53]; in fermented/dairy-analog matrices, plant–dairy blending and targeted modifications (enzymatic/physical) improve gel strength (↑G′), water-holding, and overall texture, narrowing gaps with casein networks [54]; in meat analogs, high-moisture extrusion builds anisotropic fibrous structures with tunable bite and juiciness governed by moisture, thermal–mechanical history, and mixed biopolymer systems [55]; and as a representative dairy dessert case, adding plant protein increased apparent viscosity and viscoelastic moduli while enabling fat reduction without compromising structure [25].

**Table 2:** Overview of commercial plant-based protein supplement (PBPS) product forms and their nutritional efficiency, formulation challenges, bioavailability characteristics, and application contexts.

Product Form	Nutritional Efficiency Index	Key Advantages	Limitations	Applications	Formulation Challenges and Solution	References
Protein isolates	P.C: 90–98% D: 92–97% A.R: 30–60 min	-High bioavailability -Reduced antinutrients -Excellent solubility -Minimal matrix interference	-Higher cost -Extensive processing -Potential loss of beneficial compounds	-Sports nutrition -Clinical applications -Therapeutic supplementation	-Solubility optimization via microencapsulation -Off-flavor masking via controlled processing -Oxidation prevention using antioxidant systems	[5,7]
Protein concentrates	P.C: 70–89% D: 85–92% A.R: 45–90 min	-Cost-effective -Retains beneficial fiber and micronutrients -Good functionality -Moderate processing requirements	-Lower protein density -May contain antinutrients -Variable quality between sources	-General supplementation -Food fortification -Daily nutrition enhancement	-Antinutrient reduction via enzymatic treatment -Quality standardization through controlled processing -Sensory improvement using fermentation	[1,7,13]
Protein blends	P.C: 75–90% D: 88–94% A.R: 40–80 min	-Complete amino acid profile -Nutritional synergy -Balanced functionality -Cost optimization through strategic combinations	-Complex formulation requirements -Potential ingredient interactions -Quality control challenges across multiple sources	-Comprehensive nutrition -Meal replacement -Balanced supplementation for diverse populations	-Ratio optimization using complementation principles -Compatibility testing for ingredient interactions -Stability maintenance through processing control	[5]
Beverages	P.C: 10–25% D: 90–95% A.R: 15–45 min	-Immediate bioavailability -Consumption convenience -Rapid gastric emptying -Enhanced hydration benefits	-Protein aggregation during storage -Shorter shelf life -Packaging limitations -Higher cost per protein unit	-Post-workout recovery -On-the-go nutrition -Clinical feeding applications	-Protein stability through pH buffering -Microbial safety via aseptic processing -Separation prevention using natural emulsifiers	[29]
Protein bars	P.C: 15–30% D: 80–88% A.R: 60–120 min	-Portability -Sustained protein release -Texture variety -Combined macronutrient delivery	-Matrix interference with protein digestion -Moisture migration affecting quality -Binding challenges during processing	-Convenient snacking -Endurance sports -Meal replacement for active lifestyles	-Texture optimization using plasticizers -Moisture control through barrier technologies -Binding efficiency via novel binding agents	[51,56]
Capsules/tablets	P.C: 60–80% D: 85–93% A.R: varies	-Precise dosing capability -Complete taste masking -Excellent stability -Targeted delivery potential	-Low absolute protein per unit -Dissolution requirements -Swallowing limitations for some populations	-Targeted supplementation -Therapeutic applications -Sensitive populations with taste aversions	-Release kinetics optimization through enteric coatings -Coating integrity via controlled processing -Compression optimization using co-processing techniques	[13,57,58]
Functional foods	P.C: 5–20% D: 75–85% A.R: 90–180 min	-Seamless dietary integration -Familiar consumption patterns -Enhanced nutritional value of staple foods	-Limited protein contribution per serving -Formulation complexity -Potential sensory impact on food products	-Population-wide nutrition enhancement -Specific demographic targeting -Daily protein fortification	-Matrix compatibility through protein modification -Sensory integration via processing optimization -Stability maintenance using masking technologies	[21,56]

## 4. Novel Processing Techniques of Plant-Based Protein Supplements

While conventional processing methods, including enzymatic hydrolysis, dry fractionation, and wet fractionation, have enabled large-scale synthesis of PBPSs, several challenges persist, including low digestibility, the presence of off-flavors, and incomplete amino acid profiles. Recent advancements in processing technologies have significantly enhanced the efficiency, functionality, and nutritional value of plant-based protein supplements. These innovations address inherent limitations of plant proteins, such as poor solubility, off-flavors, and reduced digestibility, thereby enabling broader application in the food industry. The production of PBPSs involves various methods that can be categorized into mechanical, chemical, enzymatic, and biotechnological techniques. Table 3 outlines key PBPS processing techniques, their effects on protein quality, and their common applications.

### 4.1. Mechanical Processing Technology

Mechanical methods serve as fundamental approaches for protein extraction by disrupting cell walls and are continuously evolving to enhance functionality and yield. Modern mechanical approaches include the following:

**High-Pressure Processing (HPP)**

HPP is a non-thermal technology that applies pressures ranging from 100 to 800 MPa to food materials. This technique modifies protein structures, thereby enhancing functional attributes such as solubility, emulsifying capacity, and texture. HPP is recognized for making notable enhancements to plant proteins, such as improved functional properties and increased digestibility, while reducing allergenicity and antinutritional factors [56]. HPP primarily affects secondary and tertiary protein structures while minimally impacting primary structure, thus maintaining overall nutritional integrity.

For instance, HPP-treated soy protein isolate (SPI), despite showing a low surface hydrophobicity, demonstrated the highest emulsifying activity index (EAI) value after 400 MPa treatment [41]. This improvement was attributed to pressure-induced splitting of SPI’s 7S components into smaller units with increased surface activity, although unfolding of 11S polypeptides could lead to aggregation, potentially affecting surface hydrophobicity.

HPP is recognized as a safe and controllable technique that produces products with almost no toxicity. It has been demonstrated to improve the functional and nutritional properties of legume proteins, particularly their techno-functional characteristics and digestibility. High hydrostatic pressure can also inactivate microorganisms, modify texture and emulsification properties, increase protein surface hydrophobicity, improve nutritional value of plant-origin proteins, and reduce allergenicity. Therefore, HPP enhances protein digestibility, emulsification, and safety without compromising nutritional value. Table 3 details these mechanical methods, their effects on protein quality, and their typical use cases.

**Ultrasound Technology (US)/Ultrasound-Assisted Extraction (UAE)**

This eco-friendly method utilizes high-intensity sound waves (typically 20–40 kHz) to induce cavitation, which involves the formation and implosion of microscopic bubbles [68]. The implosion of microbubbles generates shear forces that rupture plant tissues, facilitating protein isolation and improving solubility.

Ultrasound modifies proteins by reducing particle size and aggregation, enhancing protein recovery, and altering secondary and tertiary structures while largely preserving primary structure. UAE has demonstrated significant increases in protein yield (e.g., cowpea: from 31.78% to 58.96%) and digestibility (from 88.27% to 89.99%), along with improved solubility, emulsification, and foaming capacities [41]. Ultrasound also aids in sensory perception through protein restructuring.

However, concerns include high energy consumption, potential for protein oxidation due to ultrasound-induced radicals, and the possibility of metal contamination from the probe. Consequently, ultrasound improves extraction efficiency and protein quality, though careful control is necessary to prevent oxidative damage.

**Pulsed Electric Field (PEF)**

PEF is a non-thermal technique involving short, high-voltage electrical pulses (typically 0.1–80 kV/cm) that induce electroporation and disrupt cell membranes, enhancing mass transfer and protein extraction. For instance, moderate-intensity pulsed electric field (MIPEF) treatment of rice and pea samples demonstrated significant improvements in functional properties [56]. Specifically, gluten from samples subjected to 20,000 pulses exhibited significant improvements in solubility, foaming capacity, and water- and oil-holding capacity. Furthermore, gluten solubility nearly doubled at pH 5, while water- and oil-holding capacity increased at pH 6 [56].

PEF is considered a “green extraction method” as it primarily uses distilled water and offers homogeneous treatment by diffusing the electric field throughout cells. This method better preserves nutritional value and sensory properties of plant-based proteins while ensuring safety. PEF effectiveness is influenced by factors such as pulse shape, frequency, duration, and electric field strength. PEF also shows promise as an emulsification technique and for recovering valuable compounds from food waste. Therefore, PEF effectively improves functional properties while minimizing thermal damage.

**Microwave-Assisted Extraction (MAE)**

MAE is considered a sustainable and efficient route for protein extraction, utilizing electromagnetic radiation (typically 2.45 GHz) to heat solvents, accelerating protein diffusion and release from plant matrices. MAE can increase extraction yield (e.g., 24% for soybean protein) and protein content (44.4%). It can also significantly enhance protein solubility (16.16%) and digestibility (82.69%) compared to conventional steam heating methods [41]. Consequently, MAE represents a sustainable, rapid, and efficient method for improving protein availability.

**Ultramicronization**

This physical treatment reduces particle size to micro- and nano-scales (typically <100 μm), modifying protein functionality including dispersibility, solubility, and bioavailability. Ultramicronization enhances protein functionality through particle size reduction, increasing surface area for improved interaction with other food components.

### 4.2. Chemical Modification Technology

Chemical modification employs specific reagents to alter protein structures by introducing functional groups or cleaving peptide bonds, thereby enhancing techno-functional characteristics. Common chemical modifications include acylation, phosphorylation, deamidation, and acid–base modifications [5]. For instance, chemically modified proteins are blended with gums in egg-simulation products to improve rheological properties, mimicking the physical characteristics of eggs.

However, the critical aspect of chemical modification is that it requires careful evaluation of safety, efficiency, and economic feasibility to ensure the production of non-toxic products. It must also ensure that the modified proteins thus produced are suitable for food production with no residues of unreacted chemicals or toxic reagents in them, along with strict regulatory requirements. Therefore, chemical processing can improve the protein behavior in food systems but must comply with safety standards for consumption. As indicated in Table 3, chemical modifications improve protein solubility and structure but must be carefully monitored for food safety compliance.

### 4.3. Enzymatic Modification Technology

Enzymatic modification is a valuable approach that utilizes specific enzymes to break down complex organic materials and hydrolyze large protein molecules into smaller, more functional peptides and free amino acids [69]. Enzymatic hydrolysis is a mild and environmentally friendly technique that efficiently enhances protein extractability and bio-functionality.

Enzymatic extraction can achieve high protein yields, ranging from 86% to 95%, along with enhanced functional properties. It significantly contributes to improving protein digestibility. Certain enzymatic hydrolysis processes can degrade antinutritional factors, such as tannins, thereby enhancing overall nutritional quality and bioavailability of plant protein products [4,8]. For example, enzymatic hydrolysis using pepsin, followed by ultrafiltration, enhanced faba bean protein solubility from 24.4% to 88.8% at pH 7, and foaming capacity from 31.2% to 122.2% at pH 5 [41]. Therefore, enzymatic treatments offer targeted, efficient, and sustainable improvements in protein quality. Enzymatic hydrolysis enhances digestibility and functional properties, as outlined in Table 3.

### 4.4. Biotechnological Innovations

Beyond conventional processing, biotechnology introduces revolutionary methods for producing and functionalizing plant proteins, enhancing their versatility and appeal.

Fermentation, encompassing both traditional and precision approaches, is a widely studied biotechnological technique for enhancing plant protein quality.

**Traditional fermentation:** Microorganisms such as lactic acid bacteria are used to convert complex organic matter into simpler compounds, significantly enhancing the nutritional value, safety, and organoleptic and functional properties of products. For example, studies have shown that fermentation can improve the in vitro digestion of soy residue and that fermented soy products offer health benefits. Additionally, soy-based yogurt exhibits improved water-holding capacity and reduced syneresis during storage, while fermented soy milk has demonstrated antidiabetic potential [41].

**Precision fermentation (PF):** This cutting-edge approach utilizes synthetic biology or genetically engineered microbes to program microorganisms (such as *Bacillus* spp. bacteria, *Saccharomyces cerevisiae* or *Pichia pastoris* yeasts, or *Trichoderma* spp. filamentous fungi) to produce specific biomolecules, including proteins, fats, enzymes, or dyes. PF enables the microbial synthesis of target proteins that closely mimic animal-derived proteins, enhancing the sensory attributes of plant-based analogs [56]. For example, yeast-expressed soy leghemoglobin imparts a meat-like color and flavor to burgers. PF can also address micronutrient deficiencies by enabling the biosynthesis of vitamin B12 via co-fermentation with engineered strains in plant-based substrates [70].

Although concerns remain regarding the safety and regulatory acceptance of genetically engineered components, current analyses indicate a low risk of allergenicity or toxicity, particularly for recombinant proteins such as soy leghemoglobin. PF offers environmental advantages due to minimal land requirements and efficient resource utilization, often repurposing waste materials as substrates, and can utilize methane to produce high-protein single-cell protein (SCP) [4]. This allows for rapid, year-round production independent of climate. Nevertheless, PF faces several challenges, including consumer skepticism toward genetically modified organisms (GMOs), scalability of fermentation processes, and regulatory complexities related to food safety and labeling.

### 4.5. Emerging Technologies: AI and 3D Food Printing

**Figure 7:**
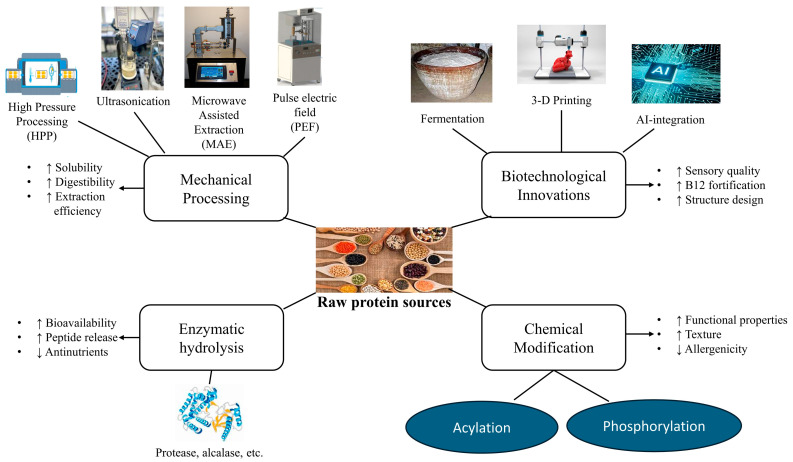
Overview of novel processing techniques for plant-based protein supplements and their functional outcomes. **Note:** ↑ indicates an increase; ↓ indicates a decrease.

Figure 7 outlines the various novel processing techniques and their functional outcomes for enhancing plant-based protein supplements.

**3D Food Printing (3DFP)/3D Extrusion:** This additive manufacturing technique uses computer-designed digital models to create food products by extruding and layering thermoplastic filaments. 3DFP enables personalized food design and the creation of intricate shapes, particularly valuable for mimicking the fibrous textures of meat in plant-based analogs [71]. For instance, fish analogs with specific porosity (15.86% in 200 filaments) can be produced from soy protein isolate (SPI) using this technique [41].

It can also create soft meals with significant applications for the elderly, such as 3D-printed hybrid meat analogs from pea protein and chicken, which show comparable moisture levels (approx. 70%) to chicken mince but reduced hardness [41]. After fermentation, 3D printing can be used to replicate the structure and texture of traditional products. Challenges include managing the mechanical qualities of printed materials and controlling moisture content. 3DFP is especially beneficial for developing dysphagia-friendly foods for elderly populations and for creating complex, structured, plant-based meat analogs [56]. Therefore, 3DFP enables personalization and structural replication of conventional foods using plant proteins.

**Artificial Intelligence (AI):** AI is revolutionizing the functional food ingredients (FFI) sector by systematically exploring and identifying safe and effective bioactive compounds. AI offers a transformative approach to ingredient characterization, enabling deeper molecular insights into food and natural product systems [72]. This enables the design of targeted ingredients to address specific health needs, such as identifying novel anti-inflammatory bioactive peptides from sources like Asian rice, leading to new functional ingredients with immunomodulatory potential.

Artificial Intelligence also helps characterize known ingredients and can accelerate the functionalization of ingredients by optimizing formulations and predicting consumer preferences. In addition, AI facilitates real-time process monitoring and supports software-driven structural optimization of proteins, which is critical for tailoring functionality in plant-based food systems [73]. While its application in characterizing plant ingredients is still developing, AI offers promising avenues for more efficient, safer, and cost-effective food solutions.

Collectively, these emerging technologies provide powerful solutions to overcome the inherent limitations of plant proteins, enabling tailored modifications that improve nutritional quality, sensory appeal, and functional performance. Their application not only enhances the competitiveness of plant-based protein products but also aligns with global goals for sustainability, food security, and personalized nutrition.

## 5. Properties and Health Benefits of Plant-Based Protein Supplements

### 5.1. Protein Quality and Nutritional Composition

**Figure 8:**
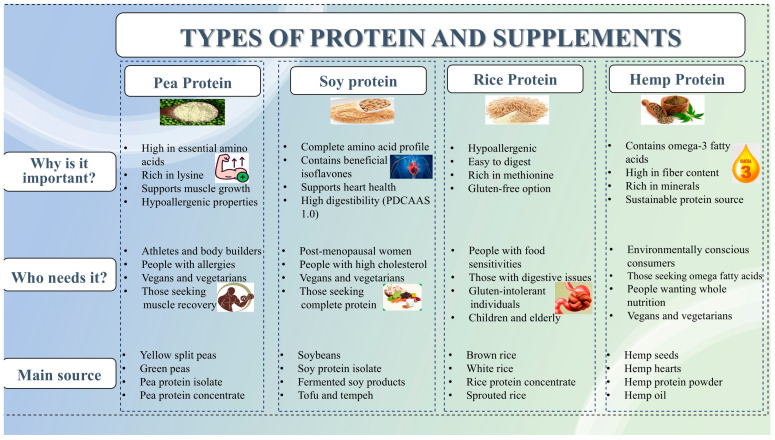
Classification of plant-based protein supplements based on source, health benefits, and target users.

The quality of a dietary protein is largely determined by its composition of indispensable (essential) amino acids (EAAs) and its digestibility. While animal-based proteins (ABPs) such as whey, casein, egg, and beef are widely recognized as complete proteins due to their high EAA content and digestibility (>95%) (depending on the source), PBPSs show significant variability in these parameters [1]. Figure 8 illustrates the classification of plant-based protein supplements based on source, health benefits, and target users.

**Amino Acid Profile**

**Figure 9:**
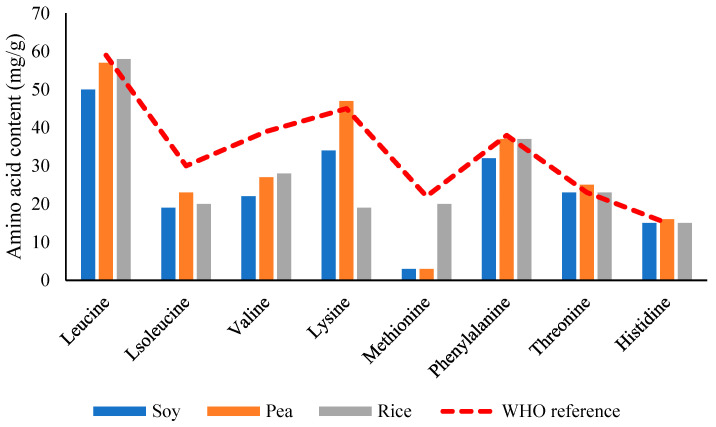
Comparative analysis of essential amino acid profiles in major plant-based protein. Refs. [57,76,77]. Note: values shown relative to WHO/FAO reference pattern for adults.

Most plant proteins are limited in one or more EAAs [5]. Legumes like peas and beans are deficient in methionine and cysteine (sulfur-containing amino acids), while cereals are often low in lysine. In contrast, proteins from sources such as soy, potato, quinoa, and canola offer favorable amino acid profiles, with PDCAAS or DIAAS values approaching or exceeding those of animal proteins. For instance, soy protein isolate (SPI) has a PDCAAS of ~1.00, comparable to milk and whey proteins [74]. As demonstrated in Figure 9, which provides a comparative illustration of essential amino acid content across various PBPSs, the strengths and limitations of each source become apparent, underscoring the importance of strategic protein selection and combination for optimal nutritional outcomes. Despite limitations in EAA content, several plant proteins are rich in conditionally indispensable amino acids. Soy protein, for instance, contains higher levels of arginine, glutamine, and glycine than whey protein. These amino acids play critical physiological roles in immune modulation, collagen synthesis, and nitrogen balance [5].

**Digestibility**

Digestibility is another key determinant of protein quality. Whole-plant proteins often show reduced digestibility (75–85%) due to complex plant matrices and the presence of antinutritional factors (ANFs), including phytates, tannins, lectins, and protease inhibitors [75]. However, modern processing techniques such as dehulling, heat treatment, enzymatic hydrolysis, fermentation, and precision extraction (e.g., ultrasound, PEF, HPP) have significantly improved the bioavailability and digestibility of plant proteins [49].

Isolated plant protein products, such as pea or soy protein isolates, often exhibit digestibility values comparable to those of animal proteins, especially when antinutrients are removed. Recent true ileal digestibility (TID) studies show that traditional plant protein foods like tofu, soy milk, and seitan can achieve TID values between 92 and 97%, approaching those of animal-based foods. Hence, protein complementation (e.g., legumes with cereals) and fortification with limiting EAAs are effective strategies to enhance the nutritional quality of PBPSs [5].

**Macronutrient Profile**

PBPSs not only provide proteins but also offer distinct macronutrient compositions that contribute to their nutritional value. Many PBPSs, especially from legumes (e.g., soybeans, peas, lentils), contain protein levels ranging from 17% to 40% (dry weight). Soy protein concentrate and isolate, for example, offer >90% protein by weight, rivaling animal-derived protein supplements [7,8].

Plant-based proteins generally contain lower saturated fat and cholesterol than animal proteins. Instead, they are rich in healthy polyunsaturated (PUFAs) and monounsaturated fatty acids (MUFAs), including omega-3 and omega-6 fatty acids [78,79]. Seeds such as flax, hemp, and chia are particularly high in essential fatty acids [56].

Additionally, PBPSs tend to be higher in complex carbohydrates and dietary fiber [5]. Legume-based supplements may include 6–62% carbohydrates and up to 27.5% fiber, promoting satiety and gut health [51]. This fiber content also impacts the glycemic response, which is generally lower for PBPSs compared to animal products. However, some commercial PBPSs may be processed with added sugars, sodium, or fats to improve palatability, potentially diminishing their natural nutritional advantages.

**Micronutrient Profile**

PBPSs offer an array of micronutrients, although their levels can vary based on the source and processing. PBPSs are good sources of certain B vitamins (except B12), vitamin E, and folate. However, they are often low in vitamin B12 and vitamin D, both predominantly found in animal products. Fortification is often necessary to bridge this gap [1,5].

Legumes and seeds are rich in iron, zinc, magnesium, and calcium; however, their bioavailability may be hindered by the presence of phytates and oxalates [80]. Nevertheless, processing techniques such as fermentation, germination, and enzymatic treatment can enhance mineral absorption by reducing these antinutritional factors [29,58,81].

Despite similar or even higher total iron content compared to meat, non-heme iron in plant foods is less bioavailable. Hence, individuals relying solely on PBPSs are advised to consume vitamin C-rich foods concurrently to improve iron absorption.

**Bioactive Compounds**

PBPSs are uniquely rich in bioactive compounds, including polyphenols, flavonoids, saponins, and phytosterols, which contribute to their health-promoting potential beyond basic nutrition.

**Figure 10:**
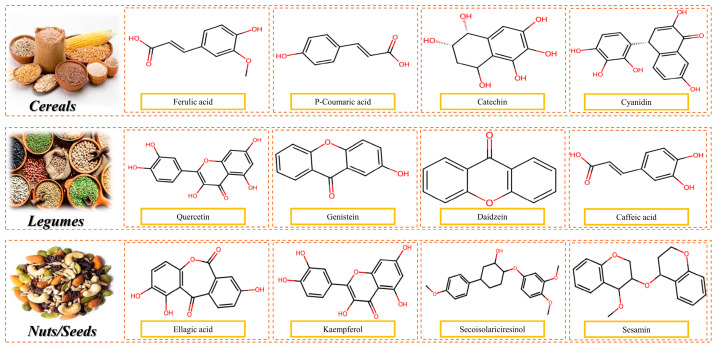
Chemical structure of common polyphenols present in cereals, legumes, and nuts and seeds.

**Polyphenols and Flavonoids:** Soy, hemp, and pumpkin seed proteins contain high concentrations of phenolic compounds, known for their antioxidant, anti-inflammatory, and anti-carcinogenic properties. These compounds also support cardiovascular health and modulate gut microbiota [4,81]. Figure 10 illustrates the chemical structure of common polyphenols present in cereals, legumes, and nuts and seeds.

**Isoflavones:** Soy protein is a rich source of isoflavones, particularly genistein and daidzein, which mimic estrogen and exhibit protective effects against hormone-dependent cancers and menopausal symptoms [1,5].

**Phytates and Saponins:** Though often labeled as antinutrients, these compounds may exert hypoglycemic, hypocholesterolemic, and anti-cancer activities at moderate levels [5].

**Bioactive Peptides:** Upon digestion or hydrolysis, plant proteins can release bioactive peptides that exhibit antihypertensive, antioxidative, antimicrobial, and antidiabetic effects [4,7,80]. Such peptides are increasingly being explored for their potential as functional food ingredients.

The diversity of these compounds positions PBPSs as promising ingredients in the development of functional foods and nutraceuticals aimed at promoting overall health and preventing chronic diseases.

### 5.2. Health Benefits of Plant-Based Protein Supplements

**Figure 11:**
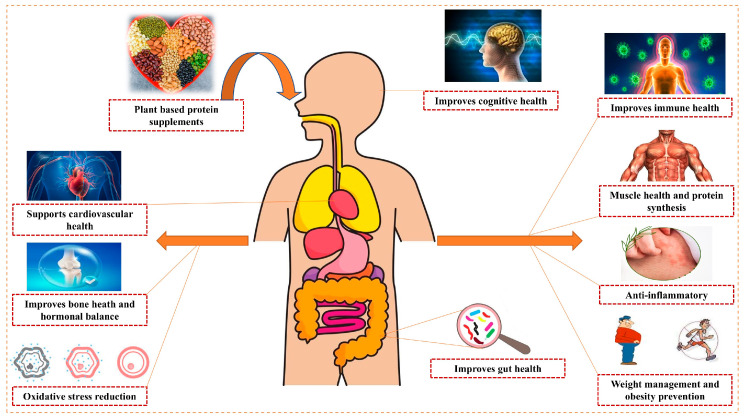
Comprehensive overview of health benefits associated with plant-based protein supplement consumption.

Figure 11 summarizes the comprehensive health benefits associated with plant-based protein supplement consumption across multiple health domains. PBPSs offer a multifaceted approach to health, muscle development, and environmental sustainability, presenting a compelling alternative to animal proteins across various dimensions. Table 4 provides a comprehensive overview of key health benefits of PBPSs, along with their mechanisms and example sources.

Beyond the general nutrient effects, PBPS benefits also involve active bio-metabolic pathways. Polyphenols in PBPSs can modulate gut microbiota composition, enhance intestinal barrier integrity (e.g., increased ZO-1 expression), and improve glycolipid metabolism [82]. Short-chain fatty acids (SCFAs), produced from dietary fibers, regulate glucose and lipid metabolism and act through mechanisms such as G-protein-coupled receptor signaling and epigenetic modifications [83]. Additionally, plant-derived peptides may inhibit angiotensin-converting enzyme (ACE), influencing blood pressure, while amino acids like leucine activate mTOR pathways, supporting muscle protein synthesis. These mechanisms underscore a synergistic bioactivity of PBPSs extending beyond basic protein provision.

**Table 4:** Health benefits of PBPSs and underlying mechanisms.

Health Domain	Key Benefits	Mechanism/Bioactive	Example Sources	Reference
Cardiovascular Health	↓ LDL and BP, ↑ Endothelial function	Arginine, polyphenols, low SFA	Soy, Pea, Flax	[81,84]
Weight Management/ Obesity Prevention	↑ Satiety, ↓ Energy intake	Fiber, protein, appetite hormones	Pea, Rice, Chia	[81]
Muscle Health and Protein Synthesis	↑ Muscle protein synthesis (MPS)	Leucine, complete AA profile	Soy, Rice + Pea Blend	[81]
Improved Glycemic Control/Type 2 Diabetes	↓ Fasting glucose, ↑ Insulin sensitivity ↑ Glycemic response	Fiber, polyphenols, DPP-4 inhibition	Lupin, Chickpea, Pea	[85]
Gut Microbiota Modulation	↑ SCFA, ↓ Inflammation	Prebiotic fiber, fermentable starches	Chia, Mycoprotein	[85,86]
Immune Function	↑ Cytokine modulation, ↓ Oxidative stress	Phycocyanin, isoflavones, lignans	Soy, Flax	[81]
Hormonal Balance (Women)	↓ Menopause symptoms, ↑ Bone health	Phytoestrogens (isoflavones)	Soy, Flax	[85]
Cognitive Support	↓ Neuroinflammation, ↑ Brain function	Omega-3, vitamin B12, peptides	Chia, Pumpkin	[85]
Oxidative Stress Reduction	↓ Oxidative stress due to ↑ antioxidant activity	Antioxidants, phenolic compounds (phenolic acids, flavonoids, ellagitannins)	Hemp	[85]
Anti-Inflammatory	↓ Inflammation in type 2 diabetes patients	Polyphenols, flavonoids	Soy, Hemp	[85]

LDL: low-density lipoprotein; BP: blood pressure; SCFA: short-chain fatty acid, ↑ indicates an increase; ↓ indicates a decrease.

**Muscle synthesis and sports nutrition**

For individuals focused on fitness and athletes, PBPSs can effectively support muscle recovery, enhance performance, and facilitate muscle growth, achieving gains in muscle mass and strength comparable to those from animal proteins, provided that the overall protein intake is sufficient. Research has demonstrated that habitual vegans and omnivores undergoing resistance training experienced similar increases in leg lean mass, muscle cross-sectional area, muscle fiber cross-sectional area, and muscle strength when both groups consumed high-protein diets [51]. However, acute studies often show that plant proteins may stimulate lower rates of muscle protein synthesis compared to animal proteins, particularly whey protein.

The effectiveness of PBPSs for muscle development depends on several factors: total protein intake (typically requiring 20–40% more plant protein than animal protein to achieve similar effects), amino acid composition (particularly leucine content), and timing of consumption. Plant-based diets contribute to recovery through their rich content of antioxidants and polyphenols, which help mitigate exercise-induced oxidative stress and inflammation [49,87]. Additionally, the typically higher carbohydrate content in plant-based eating patterns assists in replenishing glycogen stores, a vital process for sustaining athletic performance.

On the other hand, plant proteins often require leucine fortification or higher total protein intake to maximize muscle protein synthesis. Some athletes may need to consume larger volumes of food to meet protein requirements, which could affect satiety and meal timing.

**Weight management and satiety**

PBPSs contribute to satiety and support weight management through multiple mechanisms. Diets rich in plant proteins, which are typically high in fiber, can enhance satiety by slowing digestive processes and nutrient absorption, potentially leading to reduced overall calorie intake [5]. This characteristic is beneficial for maintaining healthy body weight and promoting a leaner body composition.

On the contrary, the higher fiber content, while beneficial for satiety, may cause gastrointestinal discomfort in some individuals, particularly during the adaptation period. Additionally, some commercial plant-based products may contain added sugars and fats to improve palatability, potentially negating weight management benefits.

**Cardiovascular health**

Consumption of PBPSs is strongly linked to improved cardiovascular health outcomes. Substituting animal proteins with PBPSs can decrease several risk factors for cardiovascular disease, including reductions in low-density lipoprotein (LDL) cholesterol, non-high-density lipoprotein cholesterol, and apolipoprotein B [51,80]. Studies have shown that plant protein intake, particularly soy products, can effectively lower lipid profiles in individuals with hypercholesterolemia compared to animal proteins [79]. This dietary shift also leads to a more favorable plasma lipoprotein profile by decreasing saturated fatty acid intake and increasing polyunsaturated fatty acids [48]. The bioactive compounds in plant proteins, including isoflavones in soy, contribute to these cardioprotective effects through antioxidant and anti-inflammatory mechanisms.

However, the cardiovascular benefits are most pronounced when plant proteins replace processed meats and high-saturated-fat animal products. The benefits may be less significant when compared to lean animal proteins like fish or poultry.

**Metabolic health**

PBPSs offer considerable advantages in the management of metabolic conditions like T2D and obesity. Replacing animal protein with vegetable protein has been associated with a diminished risk of developing type 2 diabetes [75]. Specific dietary interventions, such as the acute consumption of yellow pea protein, have been observed to lead to a reduction in glycemic response and overall energy intake. Similarly, beverages based on soy protein have been linked to lower postprandial glycemic responses, reduced ghrelin levels, and decreased postprandial insulin secretion [81,88].

Generally, plant-based diets result in lower body mass, blood pressure, LDL cholesterol, and HbA1c concentrations, thereby improving overall cardiometabolic profiles and lowering the risk of type 2 diabetes and associated cardiovascular morbidity and mortality. Legumes, known for their high fiber content and low glycemic index, are particularly well-suited for diabetes management [7]. Also, the metabolic benefits are most pronounced in the context of whole-food plant-based diets. Highly processed plant-based protein products may not confer the same benefits and could potentially contain added sugars that negate glycemic advantages.

**Gut health and microbiome modulation**

Plant-based dietary patterns, being inherently rich in fiber, are crucial for fostering optimal gut health and influencing the gut microbiome [81]. Abundant fiber in PBPSs helps to increase the diversity of gut microbiota, reduce gut inflammation, and potentially inhibit the growth of harmful pathogens by promoting beneficial bacteria such as Bifidobacterium and Lactobacillus species [75]. The high fiber content may cause digestive issues in some individuals, particularly those transitioning from low-fiber diets. Gradual introduction is often necessary to avoid gastrointestinal discomfort.

**Environmental and industry considerations**

From an environmental perspective, the production of PBPSs has a significantly lower impact compared to animal proteins, requiring fewer land and water resources and generating reduced greenhouse gas emissions [81]. Table 5 compares the environmental impact of PBPSs with animal-derived proteins in terms of greenhouse gas emissions (GHG), land use, and water consumption.

However, the food industry faces challenges in replicating the sensory qualities of animal products using PBPSs, which can lead to highly processed plant-based alternatives. These alternatives may have elevated levels of added salt, sugar, and fat, making their nutritional profile different from their animal counterparts; for example, plant-based cheese alternatives often contain less protein but more fat and salt than traditional cheese [7,80].

Additionally, there is a concern regarding the potential adulteration of plant-based protein powders with less expensive ingredients like whey or wheat, which could impact nutritional value and pose allergen risks if not properly labeled. Novel protein sources, such as microbial proteins or cultured meat, while offering sustainability benefits, may introduce new nutritional or safety considerations like high nucleic acid content or altered micronutrient profiles.

## 6. Challenges and Future Perspectives

### 6.1. Limitations of Plant-Based Proteins Compared to Animal Proteins

Despite growing interest in PBPSs, several challenges remain that limit their nutritional equivalence and consumer acceptance compared to animal-based proteins. These challenges can be broadly categorized into nutritional, sensory, and technological barriers.

**Nutritional challenges**

Many plant proteins are considered “incomplete” due to limiting levels of one or more indispensable amino acids (IAAs), particularly methionine, lysine, or leucine. Although processing and blending strategies can improve amino acid profiles, natural limitations in EAA content and digestibility persist. Compared to animal proteins, plant proteins generally have lower digestibility (75–85%) due to antinutritional factors such as phytates, tannins, and indigestible cell wall components. While isolates and concentrates can improve digestibility, these forms are not always present in large quantities in whole-food-based products.

To overcome these limitations, several strategies are being adopted. Blending complementary protein sources, such as cereals and legumes, provides a more balanced indispensable amino acid profile [27]. Fortification with specific amino acids—such as lysine, methionine, and leucine—has been shown to enhance both nutritional quality and muscle anabolic potential [27]. Advances in enzymatic modification and fermentation also improve digestibility and reduce antinutritional factors, thereby narrowing the gap with animal proteins [7]. Furthermore, recent functional food applications take into consideration micronutrient fortification and co-delivery systems, which can optimize bioavailability and consumer acceptance [7].

Recent studies have identified critical processing-induced limitations in plant protein functionality. Storage-related oxidation presents a major challenge, particularly for rice bran proteins where lipase-induced rancidity causes protein structural changes that compromise functional properties. The relationship between processing conditions and protein performance is complex, with moderate oxidation sometimes enhancing stability while excessive treatment leads to aggregation and reduced functionality [24].

Additionally, acute studies often report lower anabolic responses and muscle protein synthesis (MPS) after plant protein intake, although this gap can be narrowed with adequate protein quantity and strategic amino acid fortification.

**Sensory and technological barriers**

PBPSs generally face issues related to texture, flavor, and sometimes processing behavior due to earthy or beany flavors, particularly in legumes like soy. This in turn affects consumer acceptance of the product. In addition, replicating the texture, juiciness, and mouthfeel of goods derived from animals is challenging due to the inflexible functioning of many plant proteins.

Product adulteration, such as combining PBPSs with cheaper, unreported substances (such wheat or whey), is another issue that raises concerns because it compromises nutritional correctness and increases the risk of allergies. To compensate for these sensory challenges, many processed plant-based alternatives may contain higher levels of added salt, sugar, and fat, which can lead to a “health halo effect” where consumers perceive them as healthier despite potentially suboptimal nutritional profiles.

However, it is important to note that while acute protein turnover studies may suggest a lower anabolic response from plant versus animal proteins, longitudinal studies have indicated no significant difference in muscle strength and mass accrual when adequate protein intake is maintained, even with an exclusively plant-based diet.

### 6.2. Future Research Directions and Trends in the Plant-Based Protein Industry

Future research and emerging trends in the plant-based protein industry are actively focused on overcoming these limitations through various innovative strategies. To improve nutritional quality, approaches include simply consuming a greater amount of plant protein to compensate for lower IAA levels, protein complementation and blending (e.g., combining pea and brown rice protein, or cereals and legumes to achieve a more balanced amino acid profile), and amino acid fortification.

Advanced processing technologies are also crucial for enhancing digestibility, altering protein structures, and reducing ANFs. These include heat treatment, protein isolation and concentration, and non-thermal methods like high-pressure processing (HPP), which improves textural properties, solubility, and digestibility, pulsed electric field (PEF), which influences protein interactions and enhances solubility and foaming, and ultrasound (US), which modifies proteins by improving solubility and reducing particle size.

Fermentation, particularly precision fermentation, is a rapidly emerging trend, utilized to produce functional ingredients like proteins, enzymes, and vitamins such as B12, improving both nutritional quality and sensory attributes by addressing off-flavors. Research is also exploring novel protein sources beyond traditional ones, such as fungi (mycoprotein), microalgae, and insects, and developing technological advancements like 3D printing to create intricate food structures and mimic meat textures.

The application of Artificial Intelligence (AI) and Machine Learning is also being explored for ingredient discovery and optimization. AI and 3D food printing hold considerable promise for advancing plant-based protein supplements. AI-driven tools are increasingly applied in ingredient discovery and formulation optimization, enabling predictive modeling of protein functionality, digestibility, and sensory performance [94]. These approaches can shorten development timelines and reduce trial and error in blending strategies. At the same time, 3D printing technologies provide unique opportunities to overcome consumer barriers related to texture and appearance, as they allow the design of complex structures that mimic meat-like qualities or personalized nutrition formats [22,95]. Together, AI and 3D printing may bridge nutritional, sensory, and consumer acceptance gaps, accelerating the translation of plant proteins into attractive, scalable food solutions. Furthermore, future efforts emphasize holistic dietary impact studies to understand the long-term effects of whole-plant-based diets on health and the gut microbiome, as well as the critical need for consumer education and clear nutritional guidelines to help individuals make informed choices about plant-based products, recognizing that highly processed options may not always be healthier.

Consumer acceptability remains a major determinant of the success of plant-based protein supplements. While vegetarian and vegan populations have readily adopted PBPSs, non-vegetarian consumers often perceive them as inferior substitutes to animal proteins in terms of taste, mouthfeel, and satiety [96]. Negative sensory attributes such as beany or earthy flavors, along with concerns about “over-processing,” can reduce willingness to adopt these products [7]. In addition, cultural food norms and dietary traditions in many regions still strongly prioritize animal protein, creating psychological and social barriers to acceptance [27]. Trust in health benefits and transparent labeling of ingredients are also key, as consumers in non-vegetarian markets often question the authenticity and nutritional adequacy of PBPSs. Addressing these barriers through flavor masking, cleaner formulations, and targeted communication strategies will be essential for improving acceptance beyond the vegetarian niche.

## 7. Conclusions

Plant-based protein supplements (PBPSs) represent sustainable alternatives to animal proteins, meeting nutritional needs while reducing environmental impact. Advances in processing technologies and diverse protein sources enhance functional and commercial potential, though challenges in sensory quality and processing remain. Evidence shows that PBPSs can support muscle protein synthesis and offer cardiovascular, metabolic, and gut health advantages, making them valuable contributors to human health and environmental sustainability.

Future outlook and research gaps: While this review highlights technological and nutritional advances in plant-based protein supplements, several important gaps remain, despite recent progress. Long-term clinical trials are limited, and more work is needed to clarify sustained effects on muscle function, metabolic health, and gut microbiota. Integrated assessments linking sustainability metrics with consumer behavior are also scarce. Novel sources such as mycoprotein, algae, and precision-fermented proteins raise opportunities but also questions about allergenicity, regulation, and large-scale adoption. Moreover, AI-driven optimization and personalized nutrition platforms remain largely conceptual, requiring translational studies to move from models to real-world application. Addressing these areas will be essential for guiding the responsible integration of PBPSs into global food systems.

## Figures and Tables

**Table 3:** Processing methods for PBPS and their functional outcomes.

Method	Description	Impact on Protein Quality	Applications	Reference
Dry Fractionation	Mechanical separation (air classification)	Retains native structure, low yield	Pea, lentil proteins	[59]
Wet Extraction	Solubilization + isoelectric precipitation	High yield, requires solvents	Soy, rice, lupin	[59]
Ultrafiltration	Membrane-based separation	High purity, preserves bioactive	Soy isolate, hydrolyzed blends	[60]
Enzymatic Hydrolysis	Breaks protein into peptides	↑ Digestibility, ↑ solubility, ↓ allergenicity	Clinical formulas, infant nutrition	[61]
Fermentation	Microbial treatment (LAB, fungi)	↑ Bioavailability, ↓ antinutrients	Algae, soy, seed blends	[62]
Spray Drying	Converts extract to powder	Shelf-stable, cost-effective	All commercial PBPSs	[63]
Fortification	Nutrient addition (B12, Iron, Omega-3)	Targeted nutrition	Functional foods, personalized supplements	[64]
High-Pressure Processing (HPP)	Modifies protein structure via pressure	Maintains nutritional quality, ↑ functionality like gelling and emulsification	Fortified drinks, plant-based yogurts	[65]
Ultrasound	Disrupts protein structure and improves dispersion	↑ Solubility ↑ Emulsifying capacity	Soy, oats, rice proteins in emulsion	[61]
Chemical Modification (E.g., Acylation, Phosphorylation)	Alters functional group of proteins	↑ Solubility	Limited use due to safety concerns	[66]
Extrusion	Combines heat, shear, and pressure to form textured proteins	↑ Palatability	Protein snacks, meat analogs	[67]

**Note:** ↑ indicates an increase; ↓ indicates a decrease

**Table 5:** Environmental impact comparison of plant-based protein supplements vs. animal proteins.

Protein Source	GHG Emissions (kg CO_2_-eq/kg Protein)	Land Use (m^2^/kg Protein)	Water Use (L/kg Protein)	Energy Input (MJ/kg Protein)	Nitrogen Use (g N/kg Protein)
Plant-Based Proteins					
Soy protein isolate	2–2.5	16–20	2500–4000	15–25	20–35
Pea protein isolate	1.8–2.2	15–18	2000–3500	12–20	15–25
Rice protein concentrate	2.5–3	18–25	3500–5000	18–28	25–40
Hemp protein powder	1.5–2	12–16	1800–2800	10–18	12–20
Animal-Based Proteins					
Whey protein isolate	9–11	60–75	5000–7500	40–60	80–120
Beef protein (cattle)	25–35	150–200	15,000–20,000	80–120	150–250
Egg protein powder	6–8	40–55	3500–5000	35–50	60–90
Fish protein (aquaculture)	5–7	35–50	4000–6000	30–45	50–80
Ref: [89,90,91,92,93]					

## Data Availability

No new data were created or analyzed in this study. Data sharing is not applicable to this article.

## Abbreviations

The following abbreviations are used in this manuscript:

PBPS	Plant-based protein supplement
LDL	Low-density lipoprotein
CVD	Cardiovascular disease
T2D	Type 2 diabetes
PDCAAS	Protein Digestibility-Corrected Amino Acid Score
PPI	Pea protein isolate
DIAAS	Digestible Indispensable Amino Acid Score
EAI	Emulsifying activity index
SPI	Soy protein isolate
